# Germline deletions in the *EPCAM* gene as a cause of Lynch syndrome – literature review

**DOI:** 10.1186/1897-4287-11-9

**Published:** 2013-08-12

**Authors:** Katarzyna Tutlewska, Jan Lubinski, Grzegorz Kurzawski

**Affiliations:** 1 Department of Genetics and Pathology, International Hereditary Cancer Center, Pomeranian Medical University, Połabska 4, 70-115, Szczecin, Poland

**Keywords:** Lynch syndrome, *EPCAM* gene, Colon cancer, *MSH2* hypermethylation

## Abstract

Lynch syndrome (clinically referred to as HNPCC – Hereditary Non-Polyposis Colorectal Cancer) is a frequent, autosomal, dominantly-inherited cancer predisposition syndrome caused by various germline alterations that affect DNA mismatch repair genes, mainly *MLH1* and *MSH2*. Patients inheriting this predisposition are susceptible to colorectal, endometrial and other extracolonic tumors. It has recently been shown that germline deletions of the last few exons of the *EPCAM* gene are involved in the etiology of Lynch syndrome. Such constitutional mutations lead to subsequent epigenetic silencing of a neighbouring gene, here, *MSH2*, causing Lynch syndrome. Thus, deletions of the last few exons of *EPCAM* constitute a distinct class of mutations associated with HNPCC. Worldwide, several investigators have reported families with *EPCAM* 3’end deletions. The risk of colorectal cancer in carriers of *EPCAM* deletions is comparable to situations when patients are *MSH2* mutation carriers, and is associated with high expression levels of *EPCAM* in colorectal cancer stem cells. A lower risk of endometrial cancer was also reported. Until now the standard diagnostic tests for Lynch syndrome have contained analyses such as immunohistochemistry and tests for microsatellite instability of mismatch repair genes. The identification of *EPCAM* deletions or larger *EPCAM-MSH2* deletions should be included in routine mutation screening, as this has implications for cancer predisposition.

## Introduction

**Figure 1:**
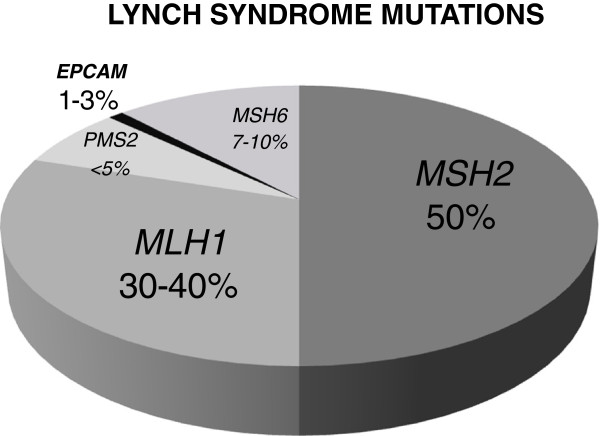
The frequency of mismatch repair gene mutations in Lynch syndrome.

Lynch Syndrome (LS; or previously HNPCC – Hereditary Non-Polyposis Colorectal Cancer) is one of the most common cancer susceptibility syndromes, which accounts for approximately 1-4% of all colon cancer cases [1]. It is characterized by an early onset of ColoRectal Cancer (CRC) and increased risk for the occurrence of several extra-colonic malignancies, in particular endometrial cancer [2]. In the largest published series 3, 1% of colorectal cases have been familiar to LS [3]. HNPCC is caused by inactivating germline mutations in the MisMatch Repair (MMR) system genes (mainly *MSH2*, *MLH1*, *MSH6*, but also *PMS2*) [4]. According to data from NCBI base *MLH1* and *MSH2* mutations account for about 90% of all mutations connected with Lynch syndrome; *MHS6* accounts for 7-10% and *PMS2* is found in less than 5% of these alterations. According to recent studies there is another gene that has an impact on Lynch syndrome (in approximately 1-3% of LS patients within the Dutch and German populations) and this is the *EPCAM* gene (Figure 1) [5].

Lynch syndrome-associated tumors are usually characterized by DNA mismatch repair deficiency, and result from a second somatic event which inactivates the remaining functional mismatch repair gene allele [6,7]. As a consequence of lack of mismatch repair, tumorigenesis is promoted by secondary mutations that accumulate at short repetitive sequences, a phenotype termed “high level microsatellite instability” (high-MSI).

In other words, a MMR gene defect in one allele gives susceptibility to further mutations which may affect second allele cause lack of mismatch repair function in cell. This results in an accumulation of mutations in coding and non-coding microsatellites in such tumors: so-called “microsatellite instability” (MSI), which is a characteristic feature of more than 95% of LS–associated CRCs [8], in addition to the loss of expression of the mutated mismatch repair gene [9]. Carriers of mutations in *MLH1, MSH2* or *MSH6* have a 30-80% cumulative risk of developing colorectal cancer and women have additional 27-71% cumulative risk of endometrial cancer below age 70 years [2]. The main clinical features are an early age of onset and the occurrence of multiple tumors.

**Figure 2:**
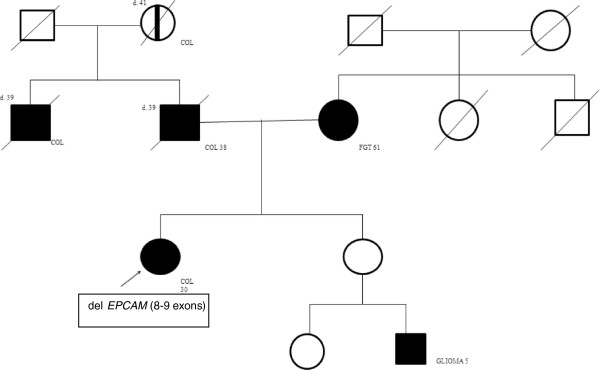
**A pedigree familiar to Lynch syndrome.** COL, colon cancer; FGT, cancer of the female genital tract; d. - age of death.

Appropriate diagnosis of LS may curried out in two major ways. One of them is to focus on an adequate family history in all patients visiting a physician. The revised Bethesda guidelines are probably the most common used criteria for selecting patients with CRC for further molecular tests [10,11] (Table 1). The other way is systematic testing for all patients with CRC for loss of MMR function by means of high level microsatellite instability in tumor tissue or immunohistochemistry (ImmunoHistoChemistry, IHC). The advantage of the immunohistochemistry, is also allowing prediction of which mismatch repair gene is likely to be affected by a germline mutation [10]. In our International Hereditary Cancer Center patients are classified to Lynch syndrome according to characteristic clinical features or criteria and pedigrees typical for Lynch syndrome, what is presented by Kladny and Lubinski [12]. An example of a pedigree of a family with definitive HNPCC and *EPCAM* carriers is shown in Figure 2.

**Table 1:** Revised Bethesda guidelines for testing colorectal tumors for microsatellite instability (MSI)

	
1	Colorectal cancer diagnosed below 50 years of age.
2	Presence of synchronous, metachronous colorectal, or other Lynch syndrome-associated* tumors, regardless of age.
3	Colorectal cancer with high-MSI*, histology diagnosed in a patient who is less than 60 years of age.
4	Colorectal cancer diagnosed with one or more first-degree relatives with Lynch syndrome – associated tumors*, with one of the cancers being diagnosed under age 50 years.
5	Colorectal cancer diagnosed in two or more first or second degree relatives with Lynch syndrome-associated tumors* regardless of age

*Lynch syndrome–associated tumors: tumors of the endometrium, small bowel, or urinary tract; high-MSI = high level microsatellite instability in tumors refers to changes in two or more of the five NCI-recommended panels of microsatellite markers.

Revised Bethesda guidelines for hereditary nonpolyposis colorectal cancer [Lynch syndrome] and microsatellite instability (Umar A, Boland CR, Terdiman JP, et al. (2004).

In some individuals with Lynch syndrome the MMR genes mutation search fails. This group is of particular interest to researchers, who trying to find the genetic factors causing the disease. In some LS patients it have been shown that MMR genes methylation cause disease occurence [13-16]. Some evidence for this came from studies in which the *MLH1* gene was the target of methylation in germline tissues in HNPCC patients who were not carriers of a germline *MLH1* mutation [17]. Moreover, heritable germline epimutations in *MSH2* have been reported as well, in some MMR germline-mutation-negative LS families [18].

A new mechanism of inactivating *MSH2* gene was therefore predicted. In multiple patients in which LS was suspected, with no germline mutation found in the MMR genes, a heterozygous germline deletion was identified encompassing the polyadenylation site located in the last two exons [8,9] of the *EPCAM* gene (OMIM#185535, formerly known as *TACSTD1*) [19]. Such deletions disrupt the 3’ end of the *EPCAM* gene, leading to transcriptional read-through of the mutated *EPCAM* allele and epigenetic inactivation, and silencing of, its neighbouring gene *MSH2*. *MSH2* is located 17 kb downstream of *EPCAM* on chromosome 2, and causes Lynch syndrome [20]. This epigenetic inactivation is restricted only to cells expressing *EPCAM*, and therefore patients who carry *EPCAM* deletions show mosaic patterns of *MSH2* inactivation that, compared with carriers of a mutation in *MSH2*, may lead to differences in tumor occurrence or spectrum [20]. What is interesting, high expression of *EPCAM* in colorectal cancer stem cells answers the question of why carriers with an *EPCAM* 3’ end deletion have a substantially increased risk of colon cancer.

Kempers and colleagues (2011) in their studies established different cancer risks associated with *EPCAM* deletions, depending on whether a deletion affects only the *EPCAM* gene or both the *EPCAM* and its neighboring gene *MSH2* (*EPCAM-MSH2*). These risks were then compared with those for Lynch syndrome carriers of a mutation in MMR genes. This was the first study that described the cumulative cancer risks and cancer profile of *EPCAM* deletion carriers [21]. They reported a low risk for endometrial cancer in patients with deletions of *EPCAM* compared to that with a mutation in an MMR gene. Of the 194 individuals with an *EPCAM* mutation included in their studies, 16 developed cancers other than colonic or endometrial [duodenal: n=3; and pancreatic: n=4; were the most common].

An interesting recent study by Kloor et al. (2011) revealed that concomitant lack of *EPCAM* and *MSH2* protein expression is a feature highly specific for cancers from *EPCAM* deletion carriers, suggesting *EPCAM* immunohistochemistry as a potential analysis tool for the identification of Lynch syndrome patients with *EPCAM* germline deletions [22]. However, there was a problem with this approach, because *EPCAM* protein expression was retained in some cancers from *EPCAM* mutation carriers. Investigators have not determined the relationship between *EPCAM* protein expression status in cancers and localization of an *EPCAM* germline deletion [22]. This is why Huth et al. (2012) hypothesized that a second somatic hit (leading to *MSH2* inactivation) determined *EPCAM* expression in tumor cells. (They analyzed four carcinomas and two adenomas from *EPCAM* deletion carriers for *EPCAM* protein expression and allelic deletion status of the *EPCAM* gene [7].

### The *EPCAM* gene

The *EPCAM* gene (Epithelial Cellular Adhesion Molecule; alternative name *TACSTD1* - Tumor Associated Calcium Signal TransDucer 1, OMIM#185535) encodes a carcinoma-associated antigen which is a glycosylated member of a family that includes at least two type I membrane proteins [23]. It is located on the short (p) arm of chromosome 2 at position 21. More precisely, from base pair 47,596,286 to base pair 47,614,166 on chromosomes 2.

**Figure 3:**
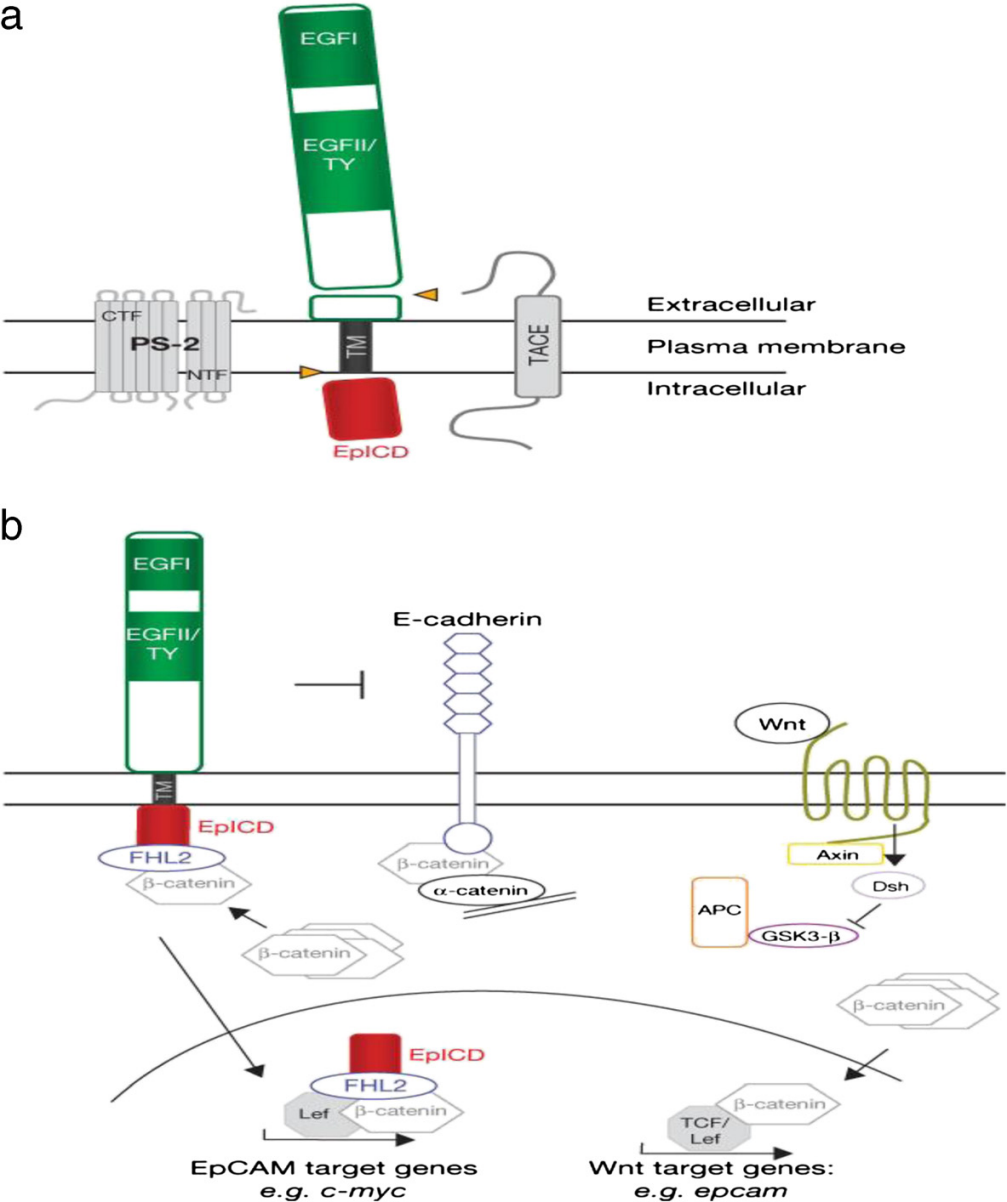
**Schematic representation of signaling pathways involving *EPCAM*, proposed by Maetzel D et al. (2009)**[23]**. (a)** Cleavage of *EPCAM* by TACE and PS-2. **(b)***EPCAM* signalling and possible cross-talk with E-cadherin and *Wnt* pathways. *EPCAM* signalling is induced by RIP, leading to EpICD nuclear translocation in a complex with FHL2 and -catenin. Within the nucleus, the EpICD complex interacts with Lef-1 and contacts DNA. Upon interference with E-cadherin, *EPCAM* may increase the availability of its interaction partner -catenin in the soluble fraction. Cross-talk with the *Wnt* pathway is conceivable at the level of -catenin and Lef-1 interactions with EpICD, and is known for induction of the *EPCAM* promoter by Tcf4 (Maetzel D, Denzel S, Mack B, et al., Nature Cell Biology 11, 162 – 171, 2009).

In healthy tissues *EPCAM* is located in the basolateral membrane but in cancer tissues this protein is homogeneously distributed on the cell surface. *EPCAM* is not only implicated in mediating epithelial-specific intercellular adhesion, but also in intracellular signaling, migration, proliferation and differentiation. The extracellular part of *EPCAM* contains an epidermal growth factor-like domain and a presumed thyroglobulin domain. Activation of *EPCAM* signaling is mediated by intra-membrane proteolysis through which the extracellular domain is shed and the intracellular domain (EpICD) is released into the cytoplasm (Figure 3). Here it becomes part of a large nuclear complex containing the transcriptional regulators β-catenin and Lef, both components of the *wnt* signaling pathway [2].

It has, therefore, been speculated that *EPCAM* on normal epithelia is sequestered and, therefore, much less accessible to antibodies than *EPCAM* in cancer tissue, where it is homogeneously distributed on the cancer cell surface [23].

The antigen *EPCAM* is presently being used as a target for immunotherapy treatment of human carcinomas.

Deletions involving the transcription termination signal of *EPCAM* are causative in 1% to 2.8% of families with Lynch syndrome. Other *EPCAM* alterations that don’t affect the transcription termination signal cause autosomal-recessive congenital tufting enteropathy [24].

### Epigenetic silencing of *MSH2*

*EPCAM* deletions cause *MSH2* gene silencing by a mechanism known as promoter hypermethylation. Additional methyl groups attached to the *MSH2* promoter reduce the expression of the *MSH2* gene, which means that less protein is produced in epithelial cells. The MSH2 protein plays an essential role in repairing mistakes in DNA, so loss of this protein prevents proper DNA repair and mistakes accumulate as the cells continue to divide. These mistakes can lead to uncontrolled cell growth and an increased risk of cancer. Lack of *EPCAM* immunostaining in MSH2-negative CRCs is indicative of *EPCAM* gene alterations, and therefore Musulen Eva and Blanco Ignacio have recommended (2013) the performance of *EPCAM* immunohistochemistry before MLPA (Multiplex Ligation-dependent Probe Amplification) analysis [4].

Van der Klift and his group (2005) first reported deletions encompassing the 3’end of *EPCAM* which did not affect the open reading frame of *MSH2*, in a cohort of LS-suspected patients [25]. Five years later a relationship between deletion of the 3′ end of *EPCAM* and inactivation of *MSH2* was independently reported by two groups. Kovacs et al. (2009) detected four different deletions encompassing the last few exons of *EPCAM*, in five families with one or more tumors exhibiting microsatellite instability and loss of the MSH2 protein [18]. At the same time, Ligtenberg et al. (2009) detected similar *EPCAM* 3′ end deletions in four Dutch families with colorectal cancer, showing high microsatellite instability and loss of the MSH2 protein, but in which no mutations in *MSH2* were found. Additionally, in these families the deletion co-segregated with the occurrence of *MSH2*-deficient tumors and, in addition, was found to lead to transcriptional read-through into the *MSH2* locus [19]. What’s more, Ligtenberg's group (2012) noticed that this transcriptional read-through induced monoallelic hypermethylation of the *MSH2* promoter present on the same allele as the 3′ end *EPCAM* deletion. Sequence analysis defined the deletion breakpoints, with a deletion of 4909 bp denoted 859-1462_*1999del from *EPCAM* cDNA. Haplotype analysis suggested that this mutation originated from a common founder. All six high-MSI tumors from these families showed methylation of the *MSH2* promoter by methylation-specific PCR and subsequent bisulfite sequencing (Table 2). These observations convincingly indicated that deletion of the 3′ end of *EPCAM* can lead to inactivation of the *MSH2* promoter and, therefore, should be considered a novel cause of Lynch syndrome [2].

**Table 2:** **Characteristics of the *EPCAM *deletions compiled by Kuiper et al., 2011 and Mur et al., 2013**

**No**	**Nomenclature**	**Population**	**Number of families**	**Deleted *EPCAM *exons**	**Size deletion (bp)**	**Breakpoint homology (bp)**	**Type of repeat element**	**Reference**
1	c.859-1462_*1999del (founder mutation)	Dutch	16	8+9	4909	6	*AluSx/ AluSq*	Ligtenberg et al., 2009 Niessen et al., 2009
2	c.491+529_*874del	German	2	5-9	11.660	25	*AluSg/ AluSg/x*	Kuiper et al., 2011
3	c.492-509_*13721del	Swiss	2	5-9	23.829	24	*AluSp/AluSg*	Van der Klift et al., 2005
4	c.555+402_*1220del	Hungarian	1	6-9	10.355	12	*AluSx/AluSx*	Kovacs et al., 2009
5	c.555+927_*14226del	Chinese/ American	3	6-9	22.836	32	*AluY/AluSc*	Ligtenberg et al., 2009
6	c.555+901_*4492del	American	2	6-9	13.128	15	*AluY/AluSx*	Kuiper et al., 2011
7	c.858+1244_*4562del	Dutch	1	8+9	9963	18	*AluSp/AluSx*	Kuiper et al., 2011
8	c.858+1211_4529del	German	1	8+9	9963	8	*AluSp/AluSx*	Kuiper et al., 2011
9	c.858+1364_*4793del_insAG	German	1	8+9	10.074	-	*AluSp/FLAM-C Alu*	Kuiper et al., 2011
10	c.858+2478_*4507del	German/ Hungarian	3	8+9	8674	14	*AluSp/AluSx*	Kovacs et al., 2009
11	c.859-2524_*10762del	Hungarian	1	8+9	14.734	15	*AluSp/AluSp*	Kuiper et al., 2011
12	c.859-353_*618del	English	1	8+9	2419	3	*AluSx/AluSg*	Kuiper et al., 2011
13	c.859-670_*530del	German	1	8+9	2648	18	*AluSx/AluSg*	Kuiper et al., 2011
14	c.859-689_*14697del	German#	4	8+9	16.834	24	*AluSx/AluSx*	Kuiper et al., 2011
15	c.859-696_*391del	Hungarian	1	8+9	6058	19	*AluSx/AluJo*	Kovacs et al., 2009
16	c.859-1682_*2116del	German	1	8+9	5246	13	*AluJb/AluSq*	Kuiper et al., 2011
17	c.859-1605_*5862del	American	1	8+9	8879	10	*AluJb/AluSq*	Kuiper et al., 2011
18	c.859-645_*10911del	American	1	8+9	13.004	14	*AluSx/AluSx*	Van der Klift et al., 2005
19	c.423-545_*3903del	German	1	4-9	16.500	7	*AluSq/AluJo*	Kuiper et al., 2011
20	c.859-1860_MSH2:646-254del	Spanish	1	8+9 and exons 1–3 of *MSH2*	28.900	3	*AluY/AluSx*	Cabornero et al., 2011
21	c.858+2568_*4596del (founder mutation)	Spanish	3	8+9	8672	32	*AluSp/AluSx*	Mur et al., 2013
22	c.858+2488_*7469del	Spanish	2	8+9	11.600	19	*AluSp/AluSp*	Mur et al., 2013
23	c.555+894_*14194del	Chinese	2	intron5-2.4 kb upstream of MSH2;	22.8 kb	6	*AluSx/AluSq*	Ligtenberg et al., 2009

German# - includes one family from unknown European origin (van der Klift et al., 2005).

Ligtenberg et al. (2009) also analyzed the family reported by Chan et al. (2006) with heritable *MSH2* promoter methylation and identified a heterozygous deletion of 22.8 kb (*EPCAM* cDNA, 555+894_+14194del) that segregated with the disease [19]. The deletion extended from intron 5 of the *EPCAM* gene to approximately 2.4 kb upstream of *MSH2*, encompassing the 3’ end of *EPCAM* and leaving the *MSH2* promoter intact. The same authors identified the same mutation in another Chinese family, where there was no evidence for a founder mutation. Analyses such as RT-PCR and methylation-specific PCR of tissue samples from affected individuals showed that methylation of *MSH2* was limited to *EPCAM*-expressing cells (Table 2).

Huth et al. (2012) reported that lack of EPCAM expression occurs in many, but not all, tumors from Lynch syndrome patients with *EPCAM* germline deletions [7]. The differences in EPCAM expression were not related to the localization of *EPCAM* germline deletions. Therefore they hypothesized that a type of second somatic hit, leading to *MSH2* inactivation during tumor development, determines EPCAM expression in the tumor cells. In four out of six tumors, investigators detected lack of EPCAM expression accompanied by biallelic deletions affecting the *EPCAM* gene. In contrast, monoallelic retention of the *EPCAM* gene was observed in the remaining two tumors with retained EPCAM protein expression. These results indicate that EPCAM expression in tumors from *EPCAM* deletion carriers depends on the localization of a second somatic hit that inactivates *MSH2*. These data demonstrate the lack of EPCAM protein expression observed in tumors from a combination of a germline mutation and second somatic hit. They also show that heterozygous *EPCAM* germline deletions are not necessarily associated with loss of EPCAM expression in tumor tissue. The detection of a somatic mutational event causing *MSH2* inactivation in one of the *EPCAM* positive tumors, explains why some tumors from *EPCAM* deletion carriers show loss of *MSH2*, but retained EPCAM expression [7].

The incidence of *EPCAM* deletions appeared to vary between populations and was found to represent at least 1-3% of the explained Lynch syndrome families. Detailed analysis of the *EPCAM* deletions revealed their range of variability as well as their *Alu*-repeat-mediated origin as a likely mechanism for these rearrangements [5]. Indeed, all *EPCAM* deletion breakpoints characterized by various authors were located within repetitive *Alu* elements (Table 2).

*Alu* elements are a family of Short Interspersed Nuclear Elements (SINE) found only in primates and comprise about 10.5% of the human genome [26]. These are mobile retrotransposable elements that also contain a recombinogenic motive, leading them to recombinational activity. However, their mobility and susceptibility to recombination is presumably tempered by host-defensive methylation of these CpG-rich elements [26]. Deletions formed through unequal *Alu*-mediated homologous recombination involve a cross-over at regions of shared sequence identity between two parental *Alu* elements located in cis in the same orientation, with loss of the loop of intervening genomic sequence during the exchange. These deletions are identifiable by signatory tracts of perfect *Alu*-derived sequence identity, overlapping or adjacent to the deletion breakpoints, such that the deletion junction cannot be mapped to a precise nucleotide. Perez-Cabornero and colleagues (2011) mapped each of the deletion breakpoints to within short stretches of fused *Alu* sequences that shared close homology to their respective parental *Alu* elements [27]. This is the first such finding to date and prompted a revisitation of the role of *Alu* elements in the causation of Lynch syndrome.

### Prevalence of *EPCAM* deletions

Worldwide, *EPCAM* deletions were found to be present in various populations from different geographic origins [18,28] (Table 2). Their prevalence was found to vary between these populations, partly because of the presence of various founder mutations [5], and to account for up to 10% of the *MSH2* inactivating mutations. All Lynch syndrome-associated tumors from *EPCAM* deletion carriers that were available for testing showed hypermethylation of the *MSH2* promoter [13]. Detailed analyses of the breakpoints of these deletions indicated that they predominantly originate from *Alu* repeat-mediated recombination events [2]. A wide variety of different deletions could be reflected in different recombination events caused by a high number of *Alu* repeats spread across this locus [9].

This situation was documented by Grandval et al. [29], namely *EPCAM* deletions in three out of seven of their patients as *de novo* mutations, which probably reflects the relatively high *Alu* repeat-mediated recombination frequency at this locus [2].

Our unpublished data from the studies of 55 patients with LS indicates that deletions of 8 and 9 exons of the *EPCAM* gene determine 7% of LS cases without MMR mutation.

### Tumor spectrum of *EPCAM* deletions

Constitutional rearrangements affecting the open reading frame of genes typically lead to constitutive inactivation of these genes, irrespective of the cell type. In contrast, in *EPCAM* 3′ end deletion carriers *MSH2* inactivation is cell type-specific, since the epigenetic silencing of *MSH2* is restricted to cells in which the *EPCAM* locus is active and transcriptional read-through occurs. The outcome of this is that carriers of *EPCAM* deletions show mosaic patterns of *MSH2* inactivation. This phenomenon involving the inactivation of one of the *EPCAM* alleles might lead to a tumor spectrum that is different from that of germline mutations directly affecting *MSH2*[2].

Some investigators have compared the cancer risk for carriers of an intragenic *MSH2* mutation, a combined *EPCAM-MSH2* deletion, and a deletion of the 3′ end of *EPCAM*. The colorectal cancer risk of *EPCAM* mutation carriers, as reflected by the mean age at diagnosis and the cumulative risk by age 70 years, was similar to that of *EPCAM-MSH2* or *MSH2* mutation carriers. In contrast, the cumulative risk of endometrial cancer by the age of 70 years was significantly lower for 3′ end *EPCAM* deletion carriers than for combined *EPCAM-MSH2* deletion carriers and *MSH2* mutation carriers (Table 3). Importantly, the comparison of the tumor risk between the *EPCAM* and *EPCAM*-*MSH2* deletion carriers indicates that the difference in endometrial cancer risk relates to the mosaic inactivation of *MSH2* and not to a constitutive loss of *EPCAM*[20]. A relatively low incidence of endometrial tumors was also observed in several other studies [30].

**Table 3:** **Heterozygous inactivation of *EPCAM *and/or *MSH2 *in colorectal and endometrial cancer risk of carriers of different germline mutations inactivating *MSH2*, elaborated by Ligtenberg et al. (2012)**

	**Gene inactivation**		**Cancer risk**	
	***EPCAM***	***MSH2***	**Colorectal**	**Endometrial**
3’ end *EPCAM* deletion	Yes	Mosaic	High	Low
*EPCAM-MSH2* deletion	Yes	Yes	High	High
Intragenic *MSH2* deletion	No	Yes	High	High

As mentioned above, the average age at onset, the risk of colorectal cancer and the tumor phenotype in *EPCAM* deletion carriers are comparable to those carrying a typical mismatch repair gene mutation in *MLH1* or *MSH2*, whereas the cumulative risk of endometrial cancer is much lower [5,20]. In *EPCAM* deletion carriers there is a relatively low risk of endometrial cancer, which is the second most prevalent Lynch syndrome-associated malignancy in carriers of a mismatch repair mutation. *EPCAM* deletion carriers will probably be more easily recognized than carriers of an *MSH6* mutation, whose colorectal cancer risk is lower with a higher age of onset [2].

In the cohort from Grandval et al. (2012), *EPCAM* deletion carriers only developed tumors of the digestive tract. Their risk to develop colorectal cancer was particularly high, only two of the 29 deletion carriers aged over 30 being unaffected [29].

### *EPCAM* founder deletions

A number of founder mutations have been identified in MMR genes, but only one affecting the *EPCAM* gene [19,31]. This 4.9 kb *EPCAM* founder deletion, thus far observed by Ligtenberg et al. in seven Dutch families, was found to be present in nine out of ten additional families from The Netherlands, but in none of the families from other geographic origins, thus confirming its founder nature (Table 2) [5]. In 2013, Mur and Pineda detected two *EPCAM* deletions: c.858+2568_*4596del (found in three families) and c.858+2488_*7469del (in two families; all five were unrelated Spanish LS families). Furthermore, they describe the *EPCAM* c.858+2568_*4596del mutation as the first reported *EPCAM* founder mutation in Spain (Table 2) [32].

In addition, six *EPCAM* deletions were identified in more than one family originating from Germany (deletions 2 and 14 in Table 2, *n* = 2 and *n* = 4, respectively), Switzerland (deletion 3 in Table 2, *n* = 2), and the United States (deletion 6 in Table 2, *n* = 2) or from multiple origins (deletions 5 and 10 in Table 2, *n* = 3) [5].

### Diagnostics of *EPCAM* 3’end deletion carriers

Mutational screening of carriers of 3’end deletions of *EPCAM* is based on matching the Amsterdam or Bethesda criteria and is associated with an MSI phenotype or loss of MMR protein expression in tumors.

Immunohistochemical analysis of MMR protein expression is a hallmark of Lynch syndrome diagnostics, but it cannot distinguish between *EPCAM* deletion carriers and *MSH2* mutation carriers [33]. The dependence of EPCAM expression on both germline and somatic alterations explains why *EPCAM* immunohistochemistry can yield inconspicuous results in a subset of tumors from *EPCAM* deletion carriers, namely if a second somatic *MSH2* inactivating hit does not affect the *EPCAM* gene [7]. Moreover, Huth and his team reported a lack of EPCAM protein expression in a colorectal adenoma, suggesting that *EPCAM* immunohistochemistry may detect *EPCAM* deletions already at a precancerous stage [7].

Germline rearrangements in the *EPCAM* gene and *MSH2* promoter methylation are detected by using multiplex ligation-dependent probe amplification (MLPA) analysis containing probes for a specific region [9,19,32]. Huth et al. (2012) revealed, following MLPA analysis, biallelic deletions affecting the *EPCAM* gene in four out of six analyzed tumors. In the remaining two tumors, no biallelic *EPCAM* deletions were observed, and the allelic profile obtained for the *EPCAM* gene region was identical in DNA isolated from tumor tissue and matched blood samples. All tumors showing biallelic deletions in the *EPCAM* gene region were negative for EPCAM protein expression, while EPCAM protein expression was retained in the tumors which retained a one normal *EPCAM* allele. These scientists also proved that the MLPA technique is applicable for the detection of heterozygous and homozygous deletions in DNA isolated from paraffin-embedded tumor tissue (when for example no blood is available) [7].

As a additional method for detecting specified deletions in the *EPCAM* gene, Ligtenberg and Kuiper (2009) used long range PCR and real-time quantitative RT-PCR. For long range PCR across the deletion, in Dutch families, a TAKARA PCR kit was used with primers on either side of the deletion. Further, they specified the deletion by direct-sequencing, using a forward primer in combination with an internal reverse primer. For the Chinese families they performed multiple long range PCRs, which yielded an aberrant amplicon suggestive of a large deletion mutation [19]. To detect fusion transcripts, a direct RT-PCR reaction or a nested-RT-PCR was performed [19,21].

Recently, Pritchard and his team from the USA (2012) notified a comprehensive and cost-effective test called “ColoSeq” that detects all classes of mutations in Lynch and polyposis syndrome genes, using solution-based targeted capture and next-generation sequencing [34]. Due to this technique they correctly identified 28/28 (100%) pathogenic mutations in *MLH1, MSH2, MSH6, PMS2, EPCAM, APC* and *MUTYH*, including single nucleotide variants (SNVs), small insertions and deletions, and large copy number variants. These scientists focused on defining the sensitivity of heterozygous variant detection because pathogenic mutations in Lynch and polyposis syndromes are almost always heterozygous, except in the *MUTYH* gene. There was 100% reproducibility of mutation detection between independent runs [34]. The Coloseq assay demonstrated at least exon-level resolution for all large deletions and duplications, which was comparable or even better than the resolution of traditional approaches to these kind of mutations analysis such as MLPA, in which exact breakpoints could not be determined, because they are commonly in *Alu* or other repetitive DNA elements [34].

## Conclusions

Based on previous worldwide results, there is a strong suggestion that implementation of *EPCAM* deletion mapping in routine diagnostics on suspected Lynch syndrome families should be considered. Some studies suggest that the frequency of *EPCAM* deletions as a cause of Lynch syndrome is up to 30% in patients with MSH2–negative tumors (from IHC results) or approximately 20% of LS patients without a mutation in MMR genes [18,22].

This underlines the importance of *EPCAM* deletions in the Lynch syndrome, as it is a more frequent cause of LS than mutations in *PMS2* or *MSH6*[33].

The frequent occurrence of somatic deletions affecting the *EPCAM* gene as a second hit in tumors from *EPCAM* deletion carriers suggests that the localization of somatic events inactivating mismatch repair genes in Lynch syndrome is not random, but related to the underlying germline mutation [7].

In conclusion, *EPCAM* 3’end deletions are a recurrent cause of Lynch syndrome, and detection should be implemented in routine Lynch syndrome diagnostics.

## Competing interests

Authors declare that they have no competing interests.

## Authors’ contributions

Conception and design: KT; drafting the manuscript: KT; critical revision for important intelectual content: GK; final approval: KT, GK, JL. All authors read and approved the final manuscript.

## References

[B1] Renkonen-Sinisalo L Sampson JR Stormorken A Tejpar S Thomas HJ Wijnen J Lubiński J Müller H Ponz De Leon M Vasen HF Möslein G Alonso A Aretz S Bernstein I Bertario L Blanco I Bulow S Burn J Capella G Colas C Engel C Frayling I Rahner N Hes FJ Hodgson S Mecklin JP Møller P Myrhøj T Nagengast FM Parc Y Recommendations to improve identification of hereditary and familial colorectal cancer in Europe Fam Cancer 2010 9 109 115 10.1007/s10689-009-9291-3 19763885

[B2] Ligtenberg MJL Kuiper RP van Kessel AG Hoogerbrugge N *EPCAM* deletion carriers constitute a unique subgroup of Lynch syndrome patients Fam Cancer 2012 10.1007/s10689-012-9591-x 10.1007/s10689-012-9591-x 23264089

[B3] Moreira L Balaguer F Lindor N de la Chapelle A Hampel H Aaltonen LA Hopper JL Le Marchand L Gallinger S Newcomb PA Haile R Thibodeau SN Gunawardena S Jenkins MA Buchanan DD Potter JD Baron JA Ahnen DJ Moreno V Andreu M Ponz De Leon M Rustgi AK Castells A EPICOLON consortium: **identification of Lynch syndrome among patients with colorectal cancer** JAMA 2012 308 1555 65 10.1001/jama.2012.13088 23073952 PMC3873721

[B4] Musulen E Blanco I Carrato C Fernandez-Figueras MT Pineda M Capella G Ariza A Usefulness of epithelial cell adhesion molecule expression in the algorithmic approach to Lynch syndrome identification Hum Pathol 2013 44 412 6 10.1016/j.humpath.2012.06.006 23026194

[B5] Kuiper RP Vissers LE Venkatachalam R Bodmer D Hoenselaar E Goossens M Haufe A Kamping E Niessen RC Hogervorst FB Gille JJ Redeker B Tops CM van Gijn ME van den Ouweland AM Rahner N Steinke V Kahl P Holinski-Feder E Morak M Kloor M Stemmler S Betz B Hutter P Bunyan DJ Syngal S Culver JO Graham T Ligtenberg MJ Recurrence and variability of germline *EPCAM* deletions in Lynch syndrome Hum Mutat 2011 32 407 14 10.1002/humu.21446 21309036

[B6] Hemminki A Peltomäki P Mecklin JP Järvinen H Salovaara R Nyström-Lahti M de la Chapelle A Aaltonen LA Loss of the wild type MLH-1 gene is a feature of hereditary nonpolyposis colorectal cancer Nature 1994 8 405 10 10.1038/ng1294-405 7894494

[B7] Huth C Kloor M Voigt A Bozukova G Evers C Gaspar H Tariverdian M Schirmacher P Doeberitz M Bläker H The molecular basis of *EPCAM* expression loss in Lynch syndrome-associated tumors Mod Pathol 2012 25 911 6 10.1038/modpathol.2012.30 22388758

[B8] Peltomäki P Lothe RA Aaltonen LA Pylkkänen L Nyström-Lahti M Seruca R David L Holm R Ryberg D Haugen A Microsatellite instability is associated with tumors that characterized the hereditary non-polyposis carcinoma syndrome Cancer Res 1993 53 5853 5 8261393

[B9] Guarinos C Castillejo A Barberá VM Pérez-Carbonell L Sánchez-Heras AB Segura A Guillén-Ponce C Martínez-Cantó A Castillejo MI Egoavil CM Jover R Payá A Alenda C Soto JL *EPCAM* Germ line deletions as causes of Lynch syndrome in Spanish patients J Mol Diagn 2010 12 765 70 10.2353/jmoldx.2010.100039 20864635 PMC2963912

[B10] Vasen HF Blanco I Aktan-Collan K Gopie JP Alonso A Aretz S Bernstein I Bertario L Burn J Capella G Colas C Engel C Frayling IM Genuardi M Heinimann K Hes FJ Hodgson SV Karagiannis JA Lalloo F Lindblom A Mecklin JP Møller P Myrhoj T Nagengast FM Parc Y Ponz De Leon M Renkonen-Sinisalo L Sampson JR Stormorken A Möslein G Revised guidelines for the clinical management of Lynch syndrome (HNPCC): recommendations by a group of European experts Gut 2013 62 812 23 10.1136/gutjnl-2012-304356 23408351 PMC3647358

[B11] Umar A Boland CR Terdiman JP Syngal S de la Chapelle A Rüschoff J Fishel R Lindor NM Burgart LJ Hamelin R Hamilton SR Hiatt RA Jass J Lindblom A Lynch HT Peltomaki P Ramsey SD Rodriguez-Bigas MA Vasen HF Hawk ET Barrett JC Freedman AN Srivastava S Revised Bethesda guidelines for hereditary nonpolyposis colorectal cancer (Lynch syndrome) and microsatellite instability J Natl Cancer Inst 2004 96 261 8 10.1093/jnci/djh034 14970275 PMC2933058

[B12] Kładny J Lubiński J Lynch syndrome (HNPCC) Hered Cancer Clin Pract 2008 6 99 102 10.1186/1897-4287-6-2-99 19804605 PMC2735811

[B13] Nagasaka T Rhees J Kloor M Gebert J Naomoto Y Boland CR Goel A Somatic hypermethylation of *MSH2* is a frequent event in Lynch syndrome colorectal cancers Cancer Res 2010 70 3098 108 10.1158/0008-5472.CAN-09-3290 20388775 PMC2856102

[B14] Suter CM Martin DI Inherited epimutation or a haplotypic basis for propensity to silence? Nat Genet 2007 39 573 17460681 10.1038/ng0507-573a

[B15] Hitchins MP Wong JJ Suthers G Suter CM Martin DI Hawkins NJ Ward RL Inheritance of a cancer-associated *MLH1* germ-line epimutation N Eng J Med 2007 356 697 705 10.1056/NEJMoa064522 17301300

[B16] Chan TL Yuen ST Kong CK Chan YW Chan AS Ng WF Tsui WY Lo MW Tam WY Li VS Leung SY Heritable germline epimutation of *MSH2* in a family with hereditary nonpolyposis colorectal cancer Nat Genet 2006 38 1178 83 10.1038/ng1866 16951683

[B17] Gazzoli I Loda M Garber J Syngal S Kolodner RD A hereditary nonpolyposis colorectal carcinoma case associated with hypermethylation of the *MLH1* gene in normal tissue and loss of heterozygosity of the unmethylated allele in the resulting microsatellite instability-high tumor Cancer Res 2002 62 3925 8 12124320

[B18] Kovacs ME Papp J Szentirmay Z Otto S Olah E Deletions removing the last exon of *TACSTD1* constitute a distinct class of mutations predisposing to Lynch syndrome Hum Mutat 2009 30 197 203 10.1002/humu.20942 19177550

[B19] Ligtenberg MJ Kuiper RP Chan TL Goossens M Hebeda KM Voorendt M Lee TY Bodmer D Hoenselaar E Hendriks-Cornelissen SJ Tsui WY Kong CK Brunner HG van Kessel AG Yuen ST van Krieken JH Leung SY Hoogerbrugge N Heritable somatic methylation and inactivation of *MSH2* in families with Lynch syndrome due to deletion of the 3’ exons of *TACSTD1* Nat Genet 2009 41 112 7 10.1038/ng.283 19098912

[B20] Kempers MJ Kuiper RP Ockeloen CW Chappuis PO Hutter P Rahner N Schackert HK Steinke V Holinski-Feder E Morak M Kloor M Büttner R Verwiel ET van Krieken JH Nagtegaal ID Goossens M van der Post RS Niessen RC Sijmons RH Kluijt I Hogervorst FB Leter EM Gille JJ Aalfs CM Redeker EJ Hes FJ Tops CM van Nesselrooij BP van Gijn ME Ligtenberg MJ Risk of colorectal and endometrial cancers in *EPCAM* deletion-positive Lynch syndrome: a cohort study Lancet Oncol 2011 12 49 55 10.1016/S1470-2045(10)70265-5 21145788 PMC3670774

[B21] Pérez-Cabornero L Infante Sanz M Velasco Sampedro E Lastra Aras E Acedo Becares A Miner Pino C Durán Domínguez M Frequency of rearrangements in Lynch syndrome cases associated with *MSH2:* characterization of a new deletion involving both *EPCAM* and the 5/ part of *MSH2* Cancer Prev Res 2011 4 1556 62 10.1158/1940-6207.CAPR-11-0080 21791569

[B22] Niessen RC Hofstra RM Westers H Ligtenberg MJ Kooi K Jager PO de Groote ML Dijkhuizen T Olderode-Berends MJ Hollema H Kleibeuker JH Sijmons RH Germline hypermethylation of *MLH1* and *EPCAM* deletions are a frequent cause of Lynch syndrome Genes Chromosomes cancer 2009 48 737 744 10.1002/gcc.20678 19455606

[B23] Munz M Baeuerle PA Gires O The emerging role of *EPCAM* in cancer and stem cell signaling Cancer Res 2009 69 5627 9 10.1158/0008-5472.CAN-09-0654 19584271

[B24] Sivagnanam M Schaible T Szigeti R Byrd RH Finegold MJ Ranganathan S Gopalakrishna GS Tatevian N Kellermayer R Further evidence for *EPCAM* as the gene for congenital tufting enteropathy Am J Med Genet 2010 152A 222 4 10.1002/ajmg.a.33186 20034091 PMC6691968

[B25] van der Klift H Wijnen J Wagner A Verkuilen P Tops C Otway R Kohonen-Corish M Vasen H Oliani C Barana D Moller P Delozier-Blanchet C Hutter P Foulkes W Lynch H Burn J Möslein G Fodde R Molecular characterization of the spectrum of genomic deletions in the mismatch repair genes *MSH2, MLH1, MSH6* and *PMS2* responsible for hereditary nonpolyposis colorectal cancer (HNPCC) Genes Chromosomes Cancer 2005 44 123 138 10.1002/gcc.20219 15942939

[B26] Jurka J Evolutionary impact of human *Alu* repetitive elements Curr Opin Genet 2004 14 603 8 10.1016/j.gde.2004.08.008 15531153

[B27] Hitchins MP Burn J *Alu* in Lynch syndrome: a danger SINE? Cancer Prev Res 2011 4 1527 30 10.1158/1940-6207.CAPR-11-0417 21972078

[B28] Lynch HT Lynch PM Lanspa SJ Snyder CL Lynch JF Boland CR Review of the Lynch syndrome: history, molecular genetics, screening, differential diagnosis and medicolegal ramifications Clin Genet 2009 76 1 18 19659756 10.1111/j.1399-0004.2009.01230.x PMC2846640

[B29] Grandval P Baert-Desurmont S Bonnet F Bronner M Buisine MP Colas C Noguchi T North MO Rey JM Tinat J Toulas C Olschwang S Colon-specific phenotype in Lynch syndrome associated with *EPCAM* deletion Clin Genet 2012 82 97 9 10.1111/j.1399-0004.2011.01826.x 22243433

[B30] Lynch HT Riegert-Johnson DL Snyder C Lynch JF Hagenkord J Boland CR Rhees J Thibodeau SN Boardman LA Davies J Kuiper RP Hoogerbrugge N Ligtenberg MJ Lynch syndrome-associated extracolonic tumors are rare in two extended families with the same *EPCAM* deletion Am J Gastroenterol 2011 106 1829 1836 10.1038/ajg.2011.203 21769135 PMC3805505

[B31] Pineda M González S Lázaro C Blanco I Capellá G Detection of genetic alterations in hereditary colorectal cancer screening Mutat Res 2010 693 19 31 10.1016/j.mrfmmm.2009.11.002 19931546

[B32] Caldés T Blanco I Soto J Capellá G Lázaro C Cajal T Mur P Pineda M Romero A Del Valle J Borràs E Canal A Navarro M Brunet J Rueda D Ramón Y Identyfication of a founder *EPCAM* deletion in Spanish Lynch syndrome families Clin Genet 2013 2013 2013 10.1111/cge.12152 10.1111/cge.12152 23530899

[B33] Kloor M Voigt AY Schackert HK Schirmacher P von Knebel DM Bläker H Analysis of *EPCAM* protein expression in diagnostics of Lynch syndrome J Clin Oncol 2011 29 223 7 10.1200/JCO.2010.32.0820 21115857

[B34] Pritchard CC Smith C Salipante SJ Lee MK Thornton AM Nord AS Gulden C Kupfer SS Swisher EM Bennett RL Novetsky AP Jarvik GP Olopade OI Goodfellow PJ King MC Tait JF Walsh T ColoSeq provides comprehensive Lynch and polyposis syndrome mutational analysis using massively parallel sequencing J Mol Diagn 2012 14 357 66 10.1016/j.jmoldx.2012.03.002 22658618 PMC3391416

